# Ancient water bottle use and polycyclic aromatic hydrocarbon (PAH) exposure among California Indians: a prehistoric health risk assessment

**DOI:** 10.1186/s12940-017-0261-1

**Published:** 2017-06-23

**Authors:** Sabrina B. Sholts, Kevin Smith, Cecilia Wallin, Trifa M. Ahmed, Sebastian K. T. S. Wärmländer

**Affiliations:** 1 Department of Anthropology, National Museum of Natural History, Smithsonian Institution, Washington, DC, USA; 2 Center for Experimental Archaeology at Davis (CEAD), Department of Anthropology, University of California, Davis, California USA; 3 Department of Biochemistry and Biophysics, Stockholm University, S-106 91 Stockholm, Sweden; 4 Department of Analytical Chemistry, Stockholm University, Stockholm, Sweden; 5 UCLA/Getty Conservation Programme, Cotsen Institute of Archaeology, UCLA, Los Angeles, California USA

**Keywords:** PAH, Naphthalene, Bitumen, Asphaltum, Public health, Environmental exposure, Ancient technology

## Abstract

**Background:**

Polycyclic aromatic hydrocarbons (PAHs) are the main toxic compounds in natural bitumen, a fossil material used by modern and ancient societies around the world. The adverse health effects of PAHs on modern humans are well established, but their health impacts on past populations are unclear. It has previously been suggested that a prehistoric health decline among the native people living on the California Channel Islands may have been related to PAH exposure. Here, we assess the potential health risks of PAH exposure from the use and manufacture of bitumen-coated water bottles by ancient California Indian societies.

**Methods:**

We replicated prehistoric bitumen-coated water bottles with traditional materials and techniques of California Indians, based on ethnographic and archaeological evidence. In order to estimate PAH exposure related to water bottle manufacture and use, we conducted controlled experiments to measure PAH contamination 1) in air during the manufacturing process and 2) in water and olive oil stored in a completed bottle for varying periods of time. Samples were analyzed with gas chromatography/mass spectrometry (GC/MS) for concentrations of the 16 PAHs identified by the US Environmental Protection Agency (EPA) as priority pollutants.

**Results:**

Eight PAHs were detected in concentrations of 1–10 μg/m^3^ in air during bottle production and 50–900 ng/L in water after 2 months of storage, ranging from two-ring (naphthalene and methylnaphthalene) to four-ring (fluoranthene) molecules. All 16 PAHs analyzed were detected in olive oil after 2 days (2 to 35 μg/kg), 2 weeks (3 to 66 μg/kg), and 2 months (5 to 140 μg/kg) of storage.

**Conclusions:**

For ancient California Indians, water stored in bitumen-coated water bottles was not a significant source of PAH exposure, but production of such bottles could have resulted in harmful airborne PAH exposure.

**Electronic supplementary material:**

The online version of this article (doi:10.1186/s12940-017-0261-1) contains supplementary material, which is available to authorized users.

## Background

Throughout human history, polycyclic aromatic hydrocarbons (PAHs) have been an ever-present health hazard. Consisting of two or more condensed aromatic benzene rings and occurring in a large number of isomers, the lipophilic PAHs are readily taken up by the human body and distributed to different body systems and tissues, including the fetus via maternal exposure [1]. Significant health problems associated with high and/or chronic levels of PAH exposure, which may vary between populations and groups [2, 3], include cancer, altered hormone levels, damage to internal organs, deficiencies in important nutrients such as vitamin A, reproductive and developmental impairments, and possibly neurodegeneration [4–13]. Given these deleterious effects, recently it has been suggested that increased tolerance of PAH exposure was an early human adaptation, providing an evolutionary advantage over other hominin species [14].

In modern human societies, the main sources of PAH exposure are related to fossil fuel processing, gasoline and diesel combustion, road paving, roofing, food processing, and tobacco smoking, as well as occasional extraordinary events such as major oil spills and wildfires [15]. In some regions biomass burning for space heating during winter is a key source of PAH exposure [16, 17]. In the ancient world PAH sources were fewer: hydrocarbons could be generated by burning organic materials, or encountered in the form of fossil bitumen (also known as asphaltum or petroleum) formed over millions of years by anaerobic decomposition of dead organisms. In world regions such as California, Mexico, and the Near East, bitumen spontaneously seeps to the Earth’s surface from certain geological formations, where people for at least 70,000 years have been collecting and using bitumen for a range of purposes, due to its adhesive, water-repellent, and decorative properties [18–24].

Bitumen is among the best evidence for exposure to persistent organic pollutants (POPs) in past human populations, given its excellent preservation in the archaeological record. Adverse effects of PAH exposure can sometimes manifest directly in the skeleton as e.g. poor bone quality [25], gross abnormalities [26], or reduced stature [9], but the actual exposure levels are generally unknown. Unlike heavy metals such as lead, which readily bioaccumulate in bone [27], PAHs are typically metabolized and eliminated from the human body within days of uptake [28]. As PAH concentrations in bone or hair do not reflect chronic exposure levels, the dose and timing of PAH exposure throughout life must therefore be estimated indirectly through other means. Earlier approaches to this problem include estimating daily doses of PAHs from traditional smoked foods [29], and presenting an exposure scenario for health risk assessment of a range of traditional native American activities and resources [30].

We previously suggested that PAH exposure from increased use of bitumen may have contributed to a prehistoric health decline of the Chumash Indians of the Santa Barbara Channel region [31], evident in ancient skeletal remains showing reduced stature, increased frequencies in dental defects of linear enamel hypoplasias, and skeletal lesions of porotic hyperostosis during the Late Holocene [32–40]. While these conditions are non-specific indicators of stress, early life exposure to PAHs has been linked with fetal growth disruption and anemia [10, 41–45], i.e. potential causes or factors of reduced skeletal size and porotic hyperostosis [46, 47]. In the Santa Barbara Channel, bitumen continuously seeps into the water from natural submarine sources, contributing to PAH exposure linked to size reduction and tissue abnormalities in local marine organisms [48, 49].

Bitumen was used for a variety of purposes by the Chumash and other native Californians, such as sealant for containers and watercraft, glue for fixing arrowheads and spear points to shafts, decoration on textiles and skin, an ingredient in ritual practices and medicinal remedies, chewing gum, and smoke-generating material for signaling [31, 50–54]. On the California Channel Islands, bitumen has been found in cultural strata between 10,000 and 7500 years old [55] and in baskets around 5000 years old [56]. For year-round inhabitants, population growth and extended droughts made bitumen-coated baskets crucial for storing limited supplies of drinking water, as pottery was not used on the islands during prehistoric times [57]. Over the subsequent millennia, the invention of the bitumen-sealed plank canoe (*tomol*) facilitated wide-ranging pelagic fishing as well as cross-channel transportation of both people and trade goods such as bitumen [57–59]. However, the potential levels of PAH exposure resulting from these cultural uses of bitumen have so far not been investigated [31].

In this study, we employed experimental archaeological methods to assess the health risks related to PAH exposure from the manufacture and use of bitumen-coated water bottles. We first used traditional materials and techniques to create water bottle replicas, based on ethnographic, ethnohistoric, and archaeological evidence. We then measured PAH concentrations in air during the manufacturing process, and in water and olive oil stored in one of the vessels for varying periods of time. Gas chromatography/mass spectrometry (GC/MS) was used to determine in all samples the concentrations of the 16 priority PAHs identified by the United States Environmental Protection Agency (EPA). The results were assessed in relation to recommended exposure levels for modern populations, and previous knowledge about health, diet, and technology in native Californian societies.

## Methods

### Water bottle replication

**Fig. 1:**
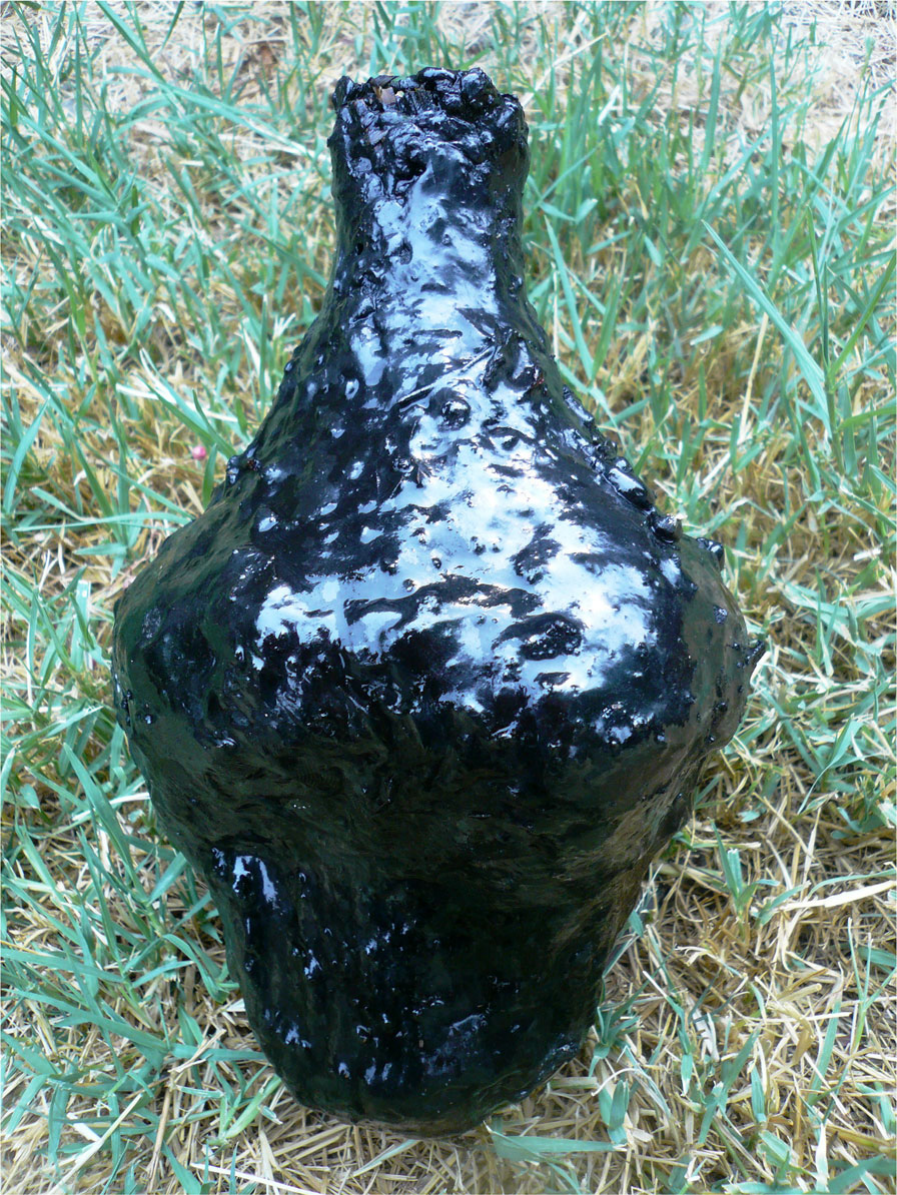
A completed water basket-bottle coated with a mixture of pitch and bitumen (*yop*), in which water and olive oil were stored for liquid PAH analysis

Two replicate water bottles (Fig. 1 and Additional file 1: Figure S1) were produced following procedures outlined in ethnohistoric accounts, and supported by archaeological finds. These replicas were manufactured in a style similar to basketry bottles from coastal and insular southern California, such as a small specimen found in the Cuyama drainage system in the Sierra Madre Mountains [60]. Using a Monterey chert flake and a bird bone awl as tools, the basketry frameworks were woven from *Juncus* sp*.* rush collected in coastal northern California, a plant previously attributed to water bottle basketry production in southern California ([60, 61]:167, 204).

The procedure for coating the twined basketry with bitumen followed ethnohistoric accounts of the Lone Woman of San Nicolas Island [62], as well as data generated from archaeological assemblages from the region [51, 56, 63]. Choosing the appropriate material(s) was however not trivial. In the Late Period (AD 1150–1782) bitumen had become a staple commodity among the Chumash, with cakes of the material “always kept on hand for immediate use” [64] and traded all along the channel shores ([65]:51–52). Different forms of the material existed. According to the ethnographic accounts collected by John P. Harrington, the historic Chumash differentiated between hard bitumen mined from land deposits (*woqo*) and soft bitumen washed ashore from submarine seeps (*malak*) ([65]:51–52)﻿. Only the hard bitumen was deemed good enough to be used when constructing the *tomol* plank canoe. Chunks of hard bitumen were then broken up with pounding stones, mixed with pine pitch and sometimes with red ochre, and boiled until the substance reached proper consistency [65]. Three different mixtures were used: the *yop*, with a larger proportion of bitumen than pitch, was used to glue the planks together; adding additional pitch to the *yop* produced a waxy substance used to waterproof the milkweed fibers that tied the planks together; and adding red ochre to the *yop* produced the mixture used as paint sealant [65]. As we did not know whether the *woqo*-bitumen or the *malak*-bitumen would be more appropriate to use for basket-coating, we made two baskets with one of each material.

For the first bottle, patches of float bitumen (*malak*) collected from the coast of San Nicolas Island in the southern California Channel Islands were used. In line with archaeological evidence [52, 63, 66], an abalone (*Haliotis rufescens*) shell was used as a mixing dish, and the *malak* was indirectly heated with tarring pebbles (Additional file 1: Figure S1 and Figure S2). These small (20–40 mm) meta-volcanic pebbles, which are virtually identical to the bitumen-coated heating stones (tarring pebbles) found in archaeological assemblages throughout the Channel Islands [56, 66], were gathered from a conglomerate outcrop on San Nicolas Island adjacent to the archaeological site of CA-SNI-40. The *malak* was then put in the base of a basketry framework, and hot tarring pebbles were added to melt the *malak*. After 1 minute, the basket was lifted with both hands and swirled rapidly in a rotary motion, generating centrifugal force that allowed the pebbles to push the *malak* into the basket weave. The process was repeated six times, after which the *malak* effectively coated the interior vessel surface. The exterior surface was coated with *malak* that was first heated with tarring pebbles in the shell dish and then applied using a beveled sea mammal bone and a piece of hard wood as tools (Additional file 1: Figure S3). After the entire bottle had been coated with *malak*, it was slowly rotated over an open flame to re-melt the *malak* on the outer surface, thereby creating a smooth exterior as well as sealing potential leaks (Additional file 1: Figure S1).

For the second bottle, hard bitumen (*woqo*) was collected from a mainland seep (“quarry”) in Goleta on the Santa Barbara coast. This site was previously an important source of bitumen for indigenous southern California groups [63]. Large chunks of solid bitumen were crushed using a hammer and anvil technique relying on two sandstone beach cobbles (Additional file 1: Figure S2). However, when this *woqo* was pulverized, it would not melt from direct heating (open flame) nor from indirect heating (hot tarring pebbles). Ethnographically, it has been noted that “high quality” mainland bitumen was mixed with conifer resin (pitch) and subsequently traded or used for gluing purposes [63]. Guided by ethnohistoric descriptions [65] and trial-and-error, we prepared a mixture consisting of roughly 55% *woqo* and 45% conifer resin from a local *Pinus radiata* species (Additional file 1: Figure S1). That is, we arrived at the mixture known to the Chumash Indians as *yop*. Our *yop* had a consistency similar to that of the *malak*, and it was applied to the basketry framework in the same way.

For the current PAH measurements, it has previously been shown that the water-soluble fraction of hydrocarbons has been washed away in *malak*/float bitumen [31, 67]. As PAH levels consequently should be higher for liquids stored in the *yop*-coated bottle, this vessel was selected for the water/oil storage experiments described below.

### Air analysis

Following standard methods developed by the US EPA in 1999, sorbent Poly-Urethane Foam (PUF) cartridges were obtained from Test America (Sacramento, California) and operated with a Gilian GilAir-5 air sampling pump from PINE Environmental (San Leandro, California). These pumps require a pre-calibration of between 1 and 5 L/min, so the pump used in this component of the study was arbitrarily set to take in air at a 3 L/min rate throughout a 30 min sample window. The pump was placed approximately 1 m from the bitumen processing area during coating of the basketry water bottle framework. The GC/MS analysis of both experimental and control (blank) samples at the TestAmerica laboratory revealed that under these experimental conditions none of the 16 targeted PAHs exceeded concentrations above the detection limits.

In a follow-up experiment, we increased the flow rate of the PUF sampler to 5 L/min (i.e. the upper limit of the pump pre-calibration) and extended the sampling period to 1 hour. Although an air flow of 7–9 L/min would be more consistent with human respiration during sedentary activities, the measured PAH concentrations (in μg/m^3^) do not depend on the air flow during sampling, and a higher air flow may run the risk of “breakthrough” where the sample media become saturated and no longer retain all pollutants collected during sampling. As shown below, the upper pre-calibration limit of 5 L/min was enough to obtain accurate concentrations for a number of PAHs. In this experimental setup we exclusively sampled the surrounding air during the process of mixing pulverized quarry bitumen and conifer resin, using the materials and techniques described above. In total, 522 g of bitumen and 422 g of conifer resin were pulverized, mixed, and heated with tarring pebbles in an abalone shell. Over the one-hour sampling period, tarring pebbles were alternated from the driftwood fire into the abalone mixing dish to keep the *yop* in a viscous state. As the experiment was conducted outdoors and a slight wind was prevailing from southwest to northeast, a 50 cm high windbreak was constructed around the bitumen processing area. For the initial 10 min of sampling, the PUF media were fixed to the wall of the windbreak and secured 40 cm above the mixing dish. During the subsequent 50 min sampling interval, the PUF media were secured approximately 10 cm above the bitumen mixing dish to ensure adequate samples of tar/pitch vapors (Additional file 1: Figure S2). Upon completion of the 1 hour field sample, the PUF media were sealed in sterile aluminum foil within an air-tight container, placed on ice to cool to around 4 °C with no post-sampling exposure to light, and transported within the hour to the TestAmerica laboratory for GC/MS analysis.

### Liquids analysis

To measure PAH concentrations in liquids stored in the bitumen-coated water bottle, controlled experiments with olive oil and distilled water were carried out at Stockholm University, Sweden. The replicate bottle was first filled with double-distilled water (ddH_2_O), which was allowed to sit in the bottle without stirring or other agitation at room temperature for a period of 2 months and then removed for analysis. The bottle was then re-filled with commercial olive oil obtained at a local super market and stored at room temperature without agitation (Additional file 1: Figure S3). Samples of the olive oil were collected after 2 days, 2 weeks, and 2 months. The water and olive oil samples were analyzed with GC/MS for the 16 EPA priority PAHs in the μg/L – ng/L range at ALS Scandinavia (Stockholm, Sweden).

## Results

For the air samples recorded during bottle manufacture, seven priority PAHs were detected in concentrations around 1–4 μg/m^3^ (Table 1). The PAHs encountered ranged from two-ring (naphthalene and methylnaphthalene) to four-ring molecules (fluoranthene). For the water sample, similar molecules were found. After 2 months of incubation in the bottle, PAHs in the size range of naphthalene to fluoranthene had accumulated in the water in concentrations of 0.05–0.9 μg/L. Also some aliphatic hydrocarbons of sizes C_16_ – C_35_ were detected (Table 1). The most abundant PAHs in the water were naphthalene, phenanthrene, and acenaphthalene, while in the air sample naphthalene, phenanthrene, and 2-methylnaphthalene showed the highest concentrations. For the olive oil stored in the bottle, all 16 PAHs tested for were encountered in concentrations above the detection limits. The concentrations of these PAHs increased over time: after 2 days of storage the PAH levels ranged from 2 to 35 μg/kg, while they ranged from 3 to 66 μg/kg after 2 weeks, and from 5 to 140 μg/kg after 2 months (Table 1). As in the water sample, the most abundant PAHs in the olive oil were two- and three-ring PAHs such as naphthalene, phenanthrene, and fluorene. The concentrations of larger four- to six-ring PAHs were an order of magnitude lower. Some PAHs were already present in the commercial olive oil before it was poured into the vessel, most notably phenanthrene (13 μg/kg), which raises questions about the quality of the product.

**Table 1:** Concentrations of individual PAHs in the air, water, and olive oil samples analyzed in this study

Compound	Air (μg/m^3^)	Water (ng/L)	Olive oil (μg/kg)			
	1 h	2 months	blank	2 days	2 weeks	2 months
Acenaphthene	1.7	37 ± 11	<0.93	1.6	4.6	7.9
Acenaphthylene	1.3	873 ± 262	<1.6	20	45	77
Anthracene	0.72	49 ± 15	3.4	9.4	9.8	25
Benzo[a]anthracene	<0.67	<0.010	2.4	6.3	6.6	12
Benzo[a]pyrene	<0.67	<0.010	<0.28	4.4	3	8
Benzo[b]fluoranthene	<0.67	<0.010	<0.6	3	4	7.8
Benzo[g,h,i]perylene	<0.67	<0.010	<0.47	2.2	3	16
Benzo[k]fluoranthene	<0.67	<0.010	<0.5	2	2.4	5.3
Chrysene	<0.67	<0.010	<0.79	3.2	7.9	13
Dibenzo[a,h]anthracene	<0.67	<0.010	<0.34	1.6	<0.79	<0.73
Fluoranthene	1.6	74 ± 22	<2.3	16	26	36
Fluorene	1.7	124 ± 37	<2.6	15	37	140
Indeno[1,2,3-cd]pyrene	<0.67	<0.010	<0.031	<0.47	<0.66	5.7
Naphthalene	4.4	206 ± 62	<12	21	31	67
Phenanthrene	4.2	281 ± 84	13	35	66	80
Pyrene	<0.67	70 ± 21	2.3	21	37	71
2-Methylnaphthalene^a^	9.2	n/a	n/a	n/a	n/a	n/a
Aliphatic C16-C35	n/a	13 ± 4	n/a	n/a	n/a	n/a

^a^Not one of the 16 priority PAHs identified by the United States Environmental Protection Agency (EPA)

## Discussion

In the global environment at least 100 different known PAHs are widespread, with typical properties such as low volatility at room temperature, poor solubility in water, and high lipophilicity. These characteristics become more pronounced with increasing molecular weight, which is accompanied by increased melting/boiling points, increased lipophilicity, decreased aqueous solubility, decreased vapor pressure, and increased resistance to oxidation and reduction (Additional file 1: Table S1). In assessing the toxic potential of an environmental sample, the 16 EPA PAHs are often used to represent all PAHs and evaluated as a sum, although other PAHs of considerably higher toxicity have been identified in the decades since the list was established [68]. The main advantage to using the 16 EPA PAHs in this study is their comparability and analytical consistency with a wide range of datasets and studies from the last several decades.

Although they are usually discussed as a group, PAHs of different molecular weight vary substantially in their behavior and distribution in the environment and the human body. Some individual PAHs, such as those detected at particularly high levels in the present study, have been linked specifically to certain effects and adverse outcomes. Most air concentration data for individual PAHs focus on the five-ring compound benzo(a)pyrene (B[a]P), which is one of the most toxic PAHs known and widely used in epidemiological studies as an indicator for PAH exposure ([69]:xx-xxi). Given that B[a]P was not detected among the 44 PAHs that we previously identified in bitumen from the California Channel Islands region [31], it is not surprising that B[a]P was also not detected in this study. Instead, the smallest and most soluble compounds (e.g., acenaphthene, fluorene, naphthalene, and phenanthrene) showed the highest concentrations in all the experimental samples (Table 1). These compounds have been assessed for toxicity relative to B[a]P, and have been assigned low toxic equivalency factors (TEFs, also known as Relative Potency Factors or RPFs) in the order of 0.001 (Additional file 1: Table S1). Using these factors, the potency of a PAH mixture can be calculated in terms of B[a]P potency equivalence (B[a]P PEQ) by the relative potency approach [70]. Each PAH concentration is then multiplied by its TEF (or RPF) factor (Additional file 1: Table S1), the contributions of each PAH are added, and the sum is the B[a]P potency equivalence which is to be compared to the EPA’s B[a]P reference values [70].

Of the detected PAHs only naphthalene — the simplest PAH, consisting of two fused benzene rings — has been given both an oral reference dose (RfD) and an inhalation reference concentration (RfC) in EPA’s Integrated Risk Information System (IRIS) (Additional file 1: Table S1). Naphthalene was furthermore one of the PAHs detected in high concentrations in all of the air, water, and oil samples. As it would be too lengthy to discuss in detail the health effects of all detected PAHs, we here focus the discussion on total PAH mixture toxicity in terms of B[a]P potency equivalence and on naphthalene as a model PAH. Naphthalene is readily absorbed by humans and other animals via inhalation, dermal contact, and oral ingestion [71]. The inhalation RfC for naphthalene is 3 × 10^−3^ mg/m^3^, calculated by the EPA based on a lowest-observed-adverse-effect-level (LOAEL) of 10 ppm (human equivalent concentration = 9.3 mg/m^3^) for nasal lesions in mice exposed by inhalation for 2 years [72]. The oral exposure RfD is 2 × 10^−2^ mg/kg*day (Additional file 1: Table S1), based on a no-observed-adverse-effect-level (NOAEL) of 100 mg/kg/day for the absence of decreased mean terminal body weight in male rats exposed by gavage for 13 weeks [72].

Air sampled while preparing *yop* for water bottle manufacture showed a moderate naphthalene concentration, i.e. 4.4 μg/m^3^ measured 10–40 cm from the melting bitumen. This is slightly higher than the EPA’s inhalation RfC of 3 μg/m^3^, i.e. the concentration of naphthalene that one supposedly can breathe every day for a lifetime that is not anticipated to cause harmful noncancer health effects. As a comparison, modern cigarette smoke measured a few meters from the cigarette contains about 2.7 μg/m^3^ of naphthalene [73], i.e. somewhat less than the bitumen smoke. As the PAHs in cigarette smoke are produced via pyrolysis, similar PAH levels would have been produced when native Californians smoked tobacco [74]. The B[a]P potency equivalence of the air sample is 140 ng/m^3^, which is much higher than EPA’s inhalation RfC of 2 ng/m^3^. The main contribution to the high B[a]P PEQ is from fluoranthene (Table 1). Given that the experimental air sample was collected very close to the melting bitumen, few individuals would have experienced such high inhalation exposure levels, at least if the bitumen was processed in an open area. Still, it cannot be ruled out that individuals who regularly worked with melted bitumen could have experienced harmful airborne PAH exposure.

Drinking water stored in the water bottle did not reach significantly high levels of PAH contamination. After 2 months of storage, the measured naphthalene concentration of 0.2 μg/L is considerably lower than the median concentration found in modern US public water systems (1 μg/L) [43]. Using the formula D = (C x IR x EF)/BW, where D = exposure dose (mg/kg*day), C = contaminant concentration (mg/L), IR = intake rate of contaminated water (L/day), EF = exposure factor (unitless), and BW = body weight (kg), the estimated exposure dose of naphthalene via drinking water ingestion for a 70 kg adult is D = (0.0002 mg/L × 3 L/day × 1)/70 kg = 8.6 × 10^−6^ mg/kg*day. Assuming that water accounted for the total fluid intake of Channel Islanders, and following the recommendation for traditional tribal communities of 3 L/day for intake rate [30], this estimate is nonetheless far below the EPA’s oral RfD for naphthalene of 2 × 10^−2^ mg/kg*day. Using the relative potency approach provides a B[a]P PEQ of 7.5 ng/L in the water, again with fluoranthene contributing most of the toxicity. Making the same assumptions as above produces an oral intake of 0.32 ng/kg*day of B[a]P PEQ, which is far less than EPA’s oral RfD for B[a]P of 0.3 μg/kg*day.

Olive oil stored in the bottle showed much higher PAH concentrations than the water, which is expected due to the general lipophilicity of PAHs (Table 1). Most PAHs showed a concentration increase over time, and olive oil stored in the bitumen-coated water bottle for 2 months showed high concentrations of in particular fluorene (140 μg/kg), phenanthrene (80 μg/kg), pyrene (71 μg/kg), and naphthalene (67 μg/kg) (Table 1). Although olives were not cultivated in California until the eighteenth century [75], these results suggest that fatty foods such as fish, shellfish, and marine mammals could have absorbed substantial amounts of PAHs from being in contact with bitumen used to e.g. coat baskets, repair fractured soapstone bowls, or glue mortar-basket hoppers [53].

Native Californians may furthermore have been exposed to PAHs by consuming marine animals from the Santa Barbara Channel, which is contaminated with PAHs from the submarine oil seeps. PAH concentrations in the Channel range from 0.6 to 28 μg/L in sediments from areas of active seepage [67, 76]. Bile naphthalene levels were between 59 and 111 μg/g in rainbow surfperch (*Hypsurus caryi*) [49] and between 5 and 20 μg/g in Pacific sanddab (*Citharichthys sordidus*) [77] caught in these waters. For California sea otters from this region the liver naphthalene levels were less than 1 μg/g [78], i.e. lower than in the fish, which is reasonable given that mammals have more efficient PAH degradation mechanisms than fish. As whole-body PAH concentrations are lower than in liver and bile, consumption of e.g. 500 g/day [30] of contaminated fish/otter would result in a naphthalene intake well below EPA’s RfD value of 20 μg/kg body weight per day [43].

In summary, non-negligible PAH levels, somewhat higher than those in cigarette smoke, were present in the fumes from the melting bitumen/pitch (*yop*) mixture. Fluoranthene was here the main toxicant. These results suggest that production of bitumen-coated objects such as water bottles and canoes could have been a source of harmful PAH exposure for prehistoric California Indians. Very small amounts of PAHs were found in water stored for 2 months in a bitumen-coated water bottle, indicating such water was safe to drink. Much higher PAH concentrations were observed in olive oil stored in the vessels, suggesting that harmful PAH exposure could have originated from fatty foods that had been in contact with bitumen-containing food processing items. Fatty fishes from the Santa Barbara Channel are contaminated with PAHs from submarine oil seeps, but consumption of such fish yields PAH intakes at tolerable levels. As PAHs typically are more toxic in mixtures due to synergistic effects [79], and as PAHs are especially harmful during the early developmental stages of life (i.e. prenatal and childhood) [11, 70, 80–82], further research and risk assessments in this area would benefit from the inclusion of age-specific information and adjustment factors.

From an archaeo-technology point of view, it is interesting that *yop* and *malak* were both found to be suitable materials for water-bottle coating, while pure *woqo* was not. One implication of this finding is that the prehistoric Channel Islanders, who only had local access to *malak* washing up on the shores, were self-sufficient with respect to water bottle manufacture. Trade of *woqo* from the mainland to the Islands is however well documented in ethnographic literature [63, 65], indicating that for certain uses the harder *woqo* was superior to the softer *malak*. It can also be noted that boiling a combination of bitumen and wood pitch to a mixture with suitable working properties is a practice described by the sixteenth century German scholar Agricola in *De Re Metallica*, his famous treatise on mining and metallurgy in mediaeval Saxony [83]. Apparently many cultures using bitumen as a crafting material discovered the advantages of this mixture. Thus, the PAH exposure data reported here are likely relevant also for crafts people from other cultures preparing similar mixtures, even though the exposure levels for specific PAHs likely vary with the bitumen/pitch ratio.

## Conclusions

For ancient California Indians, water stored in bitumen-coated water bottles was not a significant source of PAH exposure, but production of such bottles could have resulted in harmful airborne PAH exposure. Consumption of PAH-contaminated fish resulted in PAH exposure at tolerable levels. Thus, sub-lethal PAH exposure remains a possible factor in the health decline over time previously observed among the prehistoric coastal Chumash [31], but further research should emphasize the increased sensitivity to PAH exposure during prenatal and childhood stages. Studies that combine experimental archaeology with toxicology are few but valuable, as they may expand temporal perspectives in human toxicology and epidemiology, and can provide a broader evolutionary context for risk assessment in the present and future.

## Additional file

Additional file 1: Table S1. Properties of the 16 priority PAHs. **Figure S1.** Photos of water bottle manufacture. **Figure S2.** Photos of bitumen processing. **Figure S3.** Photos of water bottle manufacture and experiments. (PDF 2437 kb)

## Abbreviations

B[a]P PEQ: benzo[a]pyrene potency equivalence
EPA: US Environmental Protection Agency
GC/MS: Gas chromatography/mass spectrometry
IRIS: Integrated risk information system
PAH: Polycyclic aromatic hydrocarbon
PUF: Poly-urethane foam
RfC: Inhalation reference concentration
RfD: Oral reference dose
RPF: Relative potency factor
TEF: Toxic equivalency factor
