# Supplementary material for: Ancient water bottle use and polycyclic aromatic hydrocarbon (PAH) exposure among California Indians: a prehistoric health risk assessment
Source: Environ Health. 2017 Jun 23;16:61. doi: 10.1186/s12940-017-0261-1 (PMC5481889; doi:10.1186/s12940-017-0261-1)
Supplement: Additional file 1: Table S1. — Properties of the 16 priority PAHs. Figure S1. Photos of water bottle manufacture. Figure S2. Photos of bitumen processing. Figure S3. Photos of water bottle manufacture and experiments. (PDF 2437 kb) [file 12940_2017_261_MOESM1_ESM.pdf]

Ancient water bottle use and polycyclic aromatic hydrocarbon (PAH) exposure among California Indians: a prehistoric health risk assessment

Sabrina B. Sholts, Kevin Smith, Cecilia Wallin, Trifa M. Ahmed, Sebastian K.T.S. Wärmländer

Page

Supplementary Table S1. Properties of the 16 priority PAHs S1

Supplementary Figure S1. Photos of water bottle manufacture S2

Supplementary Figure S2. Photos of bitumen processing S3

Supplementary Figure S3. Photos of water bottle manufacture and experiments S4

Supplementary Table S1. Properties of the 16 priority PAHs identified by the US Environmental Protection Agency (EPA).

Name	CAS no.	Formula	Number of rings	Solubility in H_2O at 25 C° (mg/L)	TEF[4] /RPF[5]	Oral RfD[6] (mg/kg-day)	Critical effect
Acenaphthene	[83-32-9]	$C_{12}H_{10}$	2	3.9[1]	0.001	0.06	Hepatoxicity
Acenaphthylene	[208-96-8]	$C_{12}H_8$	2	3.93[2]			
Anthracene	[120-12-7]	$C_{14}H_{10}$	3	1.29[2]	0.001	0.3	None
Benzo(a)anthracene	[56-55-3]	$C_{18}H_{12}$	4	0.0090[1]	0.2		
Benzo(a)pyrene	[50-32-8]	$C_{20}H_{12}$	5	0.00162; 0.0038[1]	1	0.0003	
Benzo(b)fluoranthene	[205-99-2]	$C_{20}H_{12}$	5	0.0015[1]	0.8		
Benzo(g,h,i)perylene	[191-24-2]	$C_{22}H_{12}$	6	0.027 (20 C°)[1]	0.009		
Benzo(k)fluoranthene	[207-08-9]	$C_{20}H_{12}$	5	0.0008[1]	0.2/0.03		
Chrysene	[218-01-9]	$C_{18}H_{12}$	4	0.00179[1]	0.1		
Dibenzo(a,h)anthracene	[215-58-7]	$C_{22}H_{14}$	5	0.00050[1]	10		
Fluoranthene	[206-44-0]	$C_{16}H_{10}$	4	0.205; 0.26[1]	0.08	0.04	Nephropathy; hematological effects
Fluorene	[86-73-7]	$C_{13}H_{10}$	3	1.68[1]	0.001	0.04	Decreased RBC, packed cell volume, hemoglobin
Indeno(1,2,3-c,d)pyrene	[193-39-5]	$C_{22}H_{12}$	6	0.00019[1]	0.1/0.07		
Naphthalene	[91-20-3]	$C_{10}H_8$	2	31[3]	0.001	0.02	Hemolytic anaemia; decreased body weight
Phenanthrene	[85-01-8]	$C_{14}H_{10}$	3	0.977; 1.18[1]	0.001		
Pyrene	[129-00-0]	$C_{16}H_{10}$	4	0.130; 0.135[1]	0.001	0.03	Kidney effects

[1] International Agency for Research on Cancer. "Some non-heterocyclic polycyclic aromatic hydrocarbons and some related exposures." IARC monographs on the evaluation of carcinogenic risks to humans 92 (2010), Appendix.
[2] Verschueren, K. Handbook of Environmental Data of Organic Chemicals. 2nd ed. New York, NY: Van Nostrand Reinhold Co. p.139 (1983).
[3] Pearlman RS et al; J Chem Ref Data 13: 555-562 (1984).
[4] TEF = Toxicity equivalency factor. Data from: Achten C, Andersson JT. Overview of Polycyclic Aromatic Compounds (PAC). *Polycyclic Aromatic Compounds*. 35(2-4):177-186 (2015).
[5] RPF = Relative potency factor. Data from: Guidance for Evaluating the Cancer Potency of Polycyclic Aromatic Hydrocarbon (PAH) Mixtures in Environmental Samples. Minnesota Department of Health, February 8, 2016 (www.health.state.mn.us). For e.g. benzo(k)fluoranthene different TEF/RPF values have been reported.
[6] Oral reference dose value (RfD). Data taken from the US EPA's online Integrated Risk Information System (IRIS: https://www.epa.gov/iris). Compounds without reference dose values (RfD) have not been assessed by the EPA.

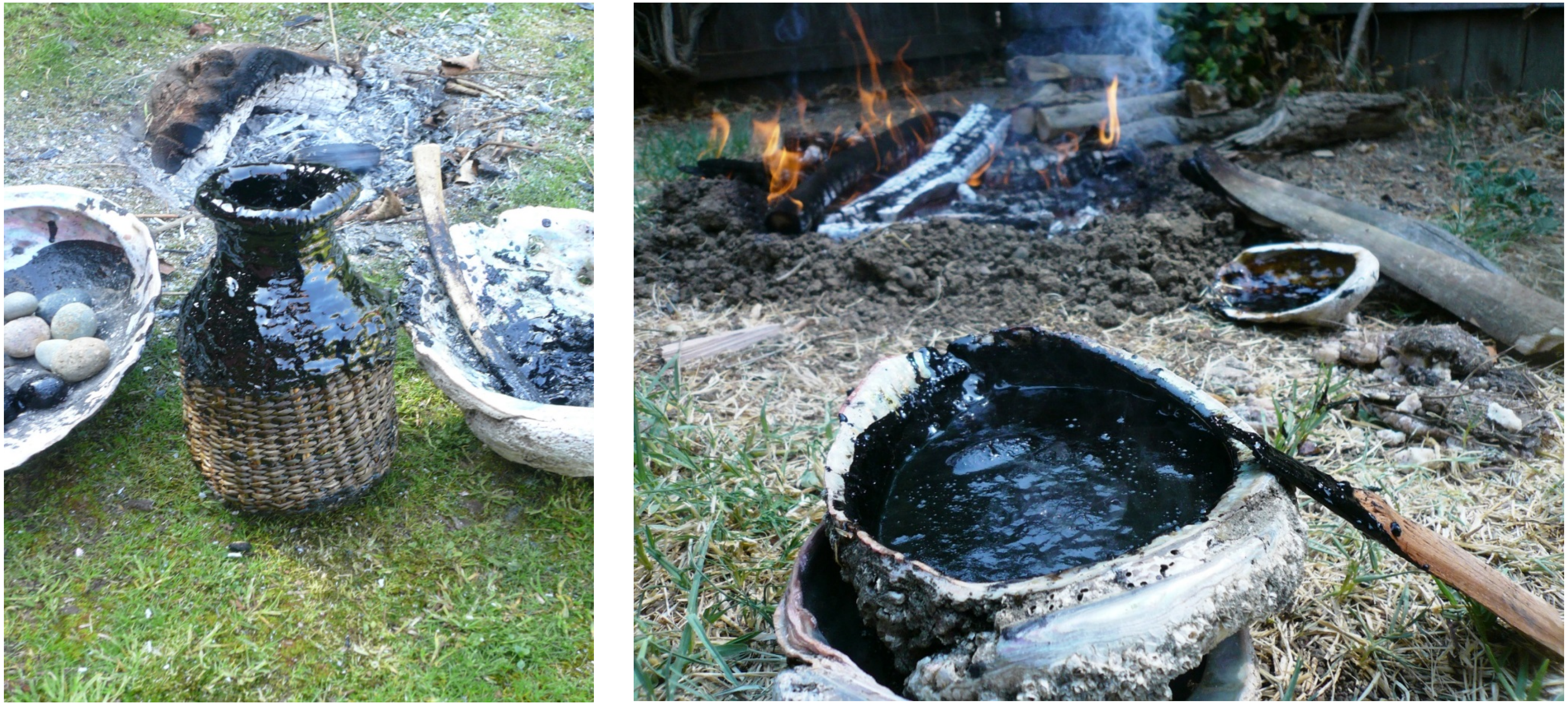

Supplementary Figure S1. Left: Twined basket framework half coated with melted float bitumen (*malak*) collected from the shoreline of San Nicolas Island. Right: Melted land bitumen (*woqo*) mixed with conifer resin, creating the *yop* mixture which has a consistency similar to that of melted float bitumen. Photos by Kevin Smith.

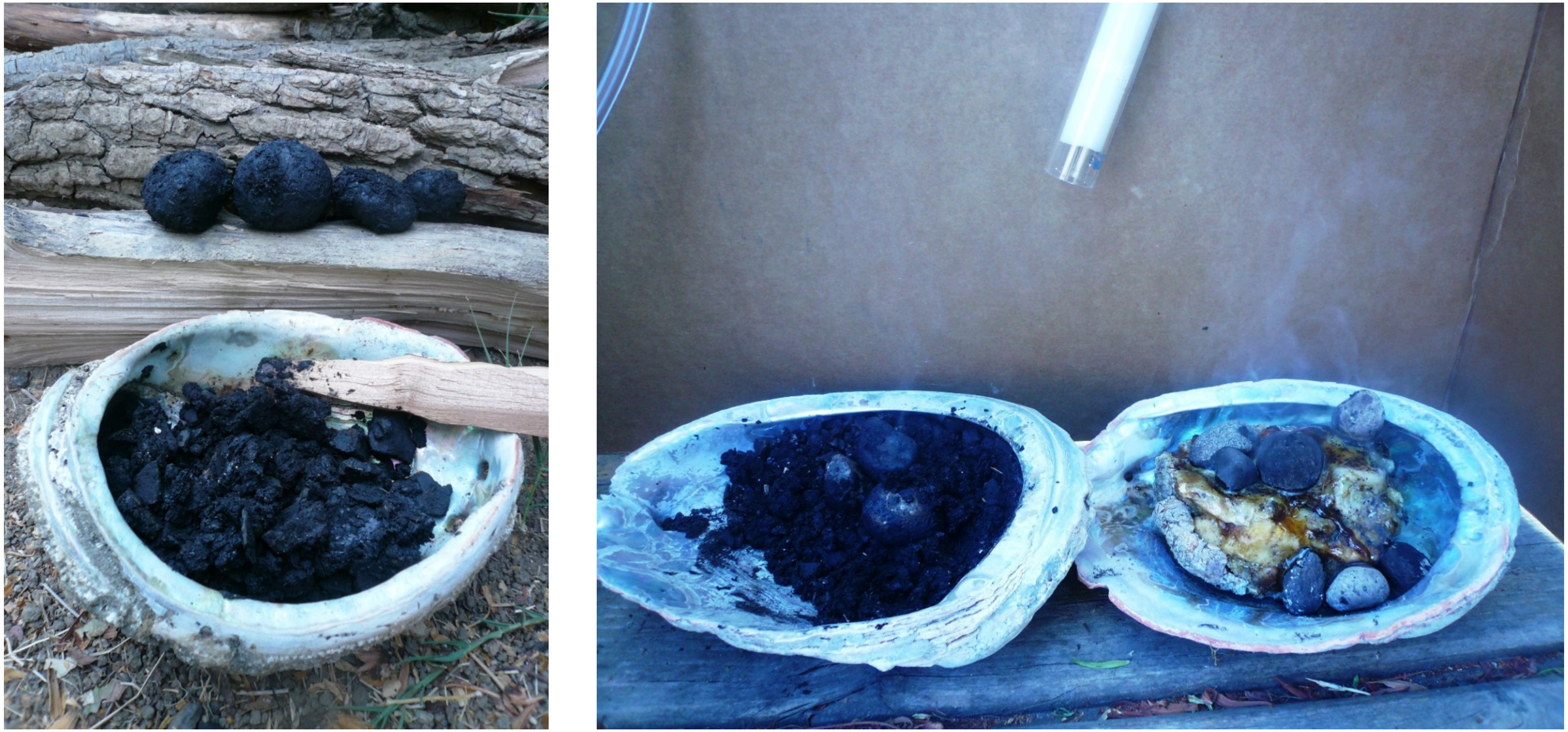

Supplementary Figure S2. Left: Bitumen collected from a terrestrial seep in Goleta, about to be melted, together with tarring pebbles used to melt the bitumen and then apply it inside the basket framework. Right: Bitumen and conifer resin heated with tarring pebbles in abalone shell mixing dishes, with the PUF media secured to the wall of the windbreak approximately 10 cm above the dishes for air analysis. Photos by Kevin Smith.

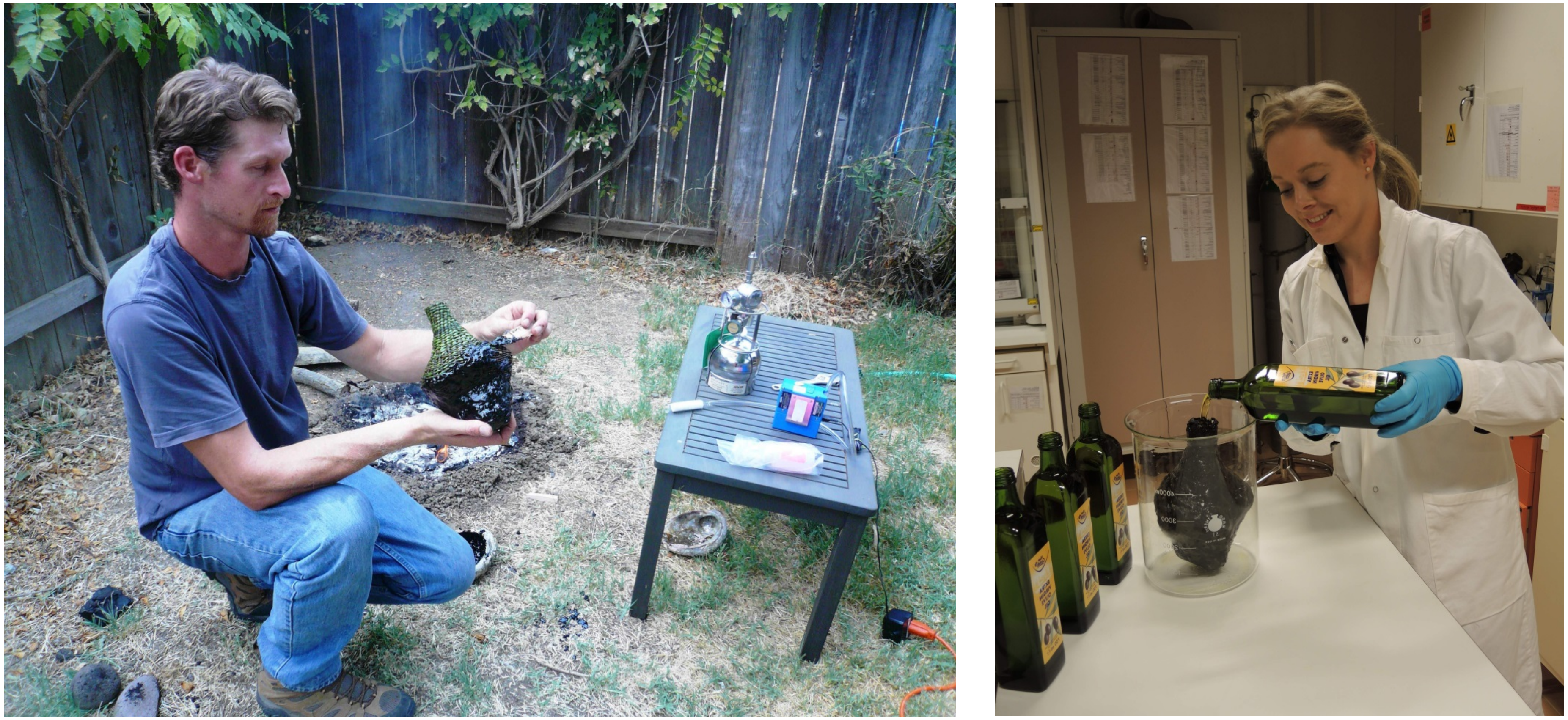

Supplementary Figure S3. Left: Kevin Smith coating the twined basketry framework with melted bitumen. Photo by Nicholas Radtkey. Right: Cecilia Wallin filling the completed bottle with commercial olive oil. Photo by Sebastian Wärmländer.
